# The need to control for regression to the mean in social psychology studies

**DOI:** 10.3389/fpsyg.2014.01574

**Published:** 2015-01-08

**Authors:** Rongjun Yu, Li Chen

**Affiliations:** ^1^Department of Psychology, School of Psychology and Center for Studies of Psychological Application, South China Normal UniversityGuangzhou, China; ^2^Scientific Laboratory of Economic Behaviors, School of Economics and Management, South China Normal UniversityGuangzhou, China

**Keywords:** regression to the mean, repeated measurements, social conformity, unrealistic optimism, social psychology

## Abstract

It is common in repeated measurements for extreme values at the first measurement to approach the mean at the subsequent measurement, a phenomenon called regression to the mean (RTM). If RTM is not fully controlled, it will lead to erroneous conclusions. The wide use of repeated measurements in social psychology creates a risk that an RTM effect will influence results. However, insufficient attention is paid to RTM in most social psychological research. Notable cases include studies on the phenomena of social conformity and unrealistic optimism (Klucharev et al., 2009, 2011; Sharot et al., 2011, 2012b; Campbell-Meiklejohn et al., 2012; Kim et al., 2012; Garrett and Sharot, 2014). In Study 1, 13 university students rated and re-rated the facial attractiveness of a series of female faces as a test of the social conformity effect (Klucharev et al., 2009). In Study 2, 15 university students estimated and re-estimated their risk of experiencing a series of adverse life events as a test of the unrealistic optimism effect (Sharot et al., 2011). Although these studies used methodologies similar to those used in earlier research, the social conformity and unrealistic optimism effects were no longer evident after controlling for RTM. Based on these findings we suggest several ways to control for the RTM effect in social psychology studies, such as adding the initial rating as a covariate in regression analysis, selecting a subset of stimuli for which the participant' initial ratings were matched across experimental conditions, and using a control group.

## Introduction

**Figure 1:**
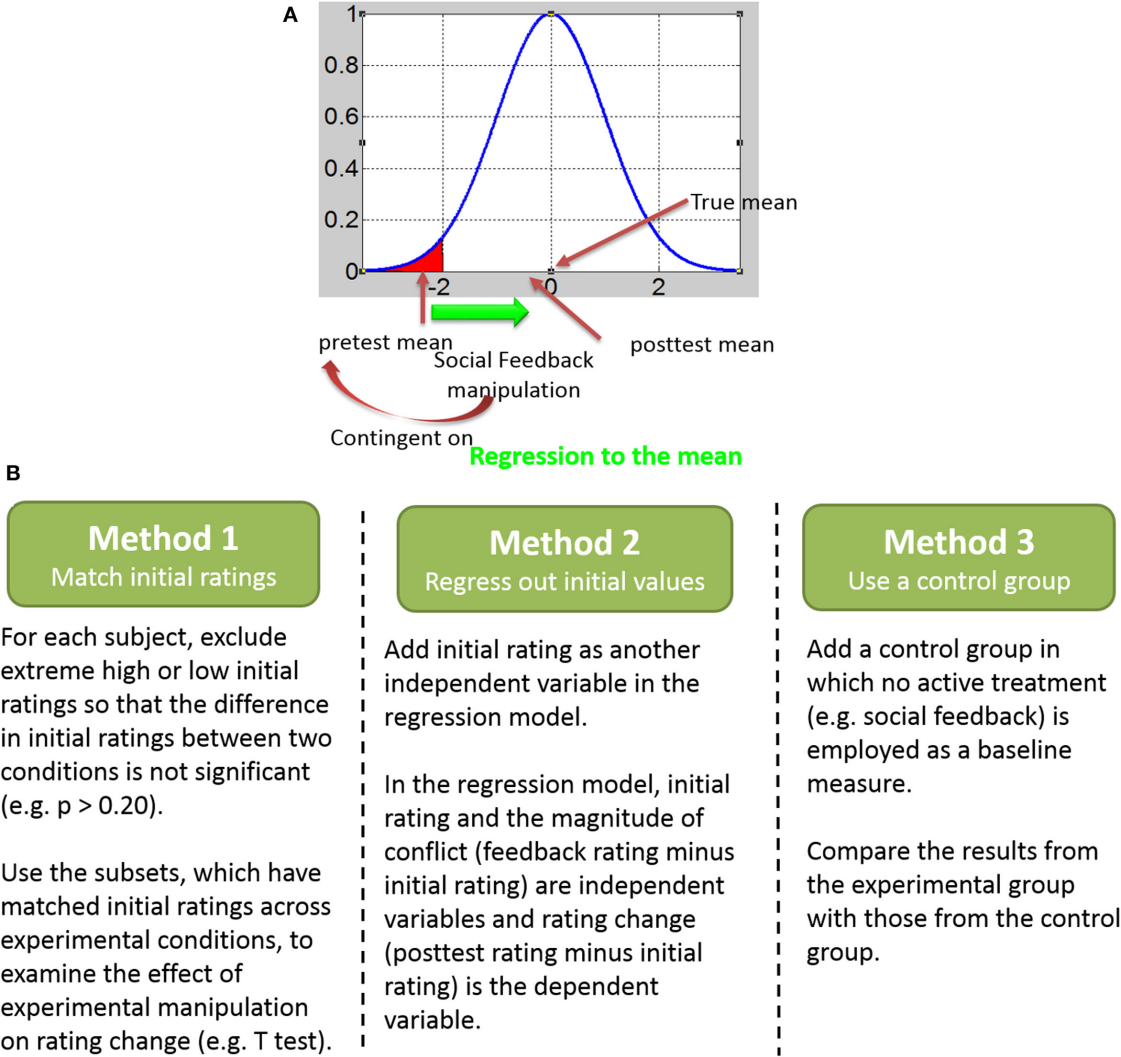
**Regression to the mean effect and ways to control for it. (A)** A graphic illustration of regression to the mean (RTM) effect. **(B)** Three ways to control for the RTM effect.

Researchers often make repeated measurements on unstable variables to obtain more accurate data or to assess change. However, measurements vary from one time point to the next due to random error, and extreme values at the first measurement tend to approach the mean at the subsequent measurement. This is known as “regression to the mean” (RTM) (Galton, 1886). The measurement of blood pressure serves as a good example. If blood pressure is initially measured in a group of patients and then re-measured after a period of time, people with extreme blood pressure at Time 1 will tend to be closer to the average level at Time 2, due to random error. RTM may be a possible explanation for the observed change since it can make natural variation seem like real change (Barnett et al., 2005). In medicine, a placebo control group in a controlled trial is usually introduced to remove the effect of RTM. People usually seek treatment when their symptoms are particularly severe. If treatment is sought when these symptoms are at their worst, these symptoms should be less severe simply by random fluctuations and natural recovery, even when no treatment is used (See Figure 1A). Thus, any treatment introduced when the symptoms are most severe will almost always lead to a coincidental recovery, even if the treatment has no effectiveness whatsoever. The placebo group which uses an inert treatment also experiences a tendency to regress to the mean. If the treatment group shows a statistically significant increase in the speed that symptoms regress, then it can be attributed to the effects of the treatment, not the placebo effect or RTM. Similarly, if a large group of students is given a test of some sort and the top-performing 10% students are selected, these people would be likely to score worse, on average, if re-tested. This is because their performance in a single test reflects individuals' true skill plus some luck rather than their normal level ability in most circumstances. Similarly, the bottom 10% would be likely to score better on a retest. In either case, the extremes of the distribution are likely to regress toward the mean due to natural random variation in the results and simple luck (you would not always be lucky or unlucky). In research, an important consequence is that experimental effects can be misleading. This has been recognized by many clinical researchers as treatments that appear to be efficacious may not show evidence of efficacy once RTM is controlled (Whitney and Von Korff, 1992; Cummings et al., 2000; Morton and Torgerson, 2003).

The wide use of repeated measurements in social psychology creates a risk that an RTM effect will influence results. However, insufficient attention is paid to RTM in most social psychological research. Notable cases include studies on the phenomena of social conformity and unrealistic optimism (Klucharev et al., 2009, 2011; Sharot et al., 2011, 2012b; Campbell-Meiklejohn et al., 2012; Kim et al., 2012; Garrett and Sharot, 2014). *Social conformity* refers to people habitually conforming to group behavior when making decisions and judgments (Asch, 1951; Festinger, 1954; Cialdini and Goldstein, 2004; Campbell-Meiklejohn et al., 2010); *unrealistic optimism* describes the tendency of people to overestimate the likelihood of future positive events and underestimate that of future negative events (Weinstein, 1980; Taylor and Brown, 1988; Armor and Taylor, 2002; Puri and Robinson, 2007; Carver et al., 2010). In recent years, studies using repeated measurements have documented both types of effects. In those studies, researchers examined social conformity by asking participants to rate the attractiveness of faces and then providing information about the group rating (challenging information) before the participants rated the faces a second time (Klucharev et al., 2009; Kim et al., 2012). In studies on the unrealistic optimism effect, participants were instructed to estimate the likelihood of experiencing adverse events and were then presented with the average probability of each event (challenging information) before again making likelihood estimation (Sharot et al., 2011, 2012b; Garrett and Sharot, 2014). In most of these studies, RTM was not recognized and taken into account in comparisons of Time 1 and Time 2 responses.

In fact, the lack of control for RTM in recent research on social conformity and unrealistic optimism is very likely to have affected the results and conclusions of these studies. In the above-cited studies on social conformity (Klucharev et al., 2009; Kim et al., 2012), participants rated facial attractiveness on an eight- or nine-point scale and the group rating that served as feedback was assigned to be above or below the participants' rating by 1, 2, or 3 points. Finally, researchers examined the social conformity effect by applying One-Way repeated measures ANOVA (with conflict between participants' initial rating and group rating as the within-subject factor: 0, ±1; ±2; and changes between the initial rating and re-rating as the dependent variable). However, as the feedback algorithm was constrained, faces with high initial ratings given by participants were likely to be assigned to the peer-lower condition (group rating was lower than participants' rating) and faces with low initial ratings given by participants were likely to be assigned to the peer-higher condition (group rating was higher than participants' rating) (Huang et al., 2014). This means that later, in the re-rating session, high initial ratings in the peer-lower condition as well as low initial ratings in the peer-higher condition might have tended to approach the average. Thus, the apparent social conformity effect may have been influenced by RTM. In the Klucharev et al. (2009) study the researchers did in fact test for the RTM effect by comparing the variances of ratings given by participants with either a central or a more extreme response tendency. This was an important step in recognizing the influence of RTM in social psychological research. However, this approach does not fully control for the RTM effect, as extreme ratings at the initial rating session may still regress to the mean at the re-rating session.

Recent studies on unrealistic optimism (Sharot et al., 2011, 2012b; Garrett and Sharot, 2014) also are problematic. Although the research designs in these studies made RTM likely, RTM was not assessed or controlled for. In these studies, participants were asked to make estimations of adverse life events. To make the range of possible underestimation equal to that of possible overestimation, participants were told that the range of average probabilities (presented as challenging information) was between 3% and 77% while it actually lay between 10% and 70%. As a consequence, events with a high estimation were more likely to be assigned to the desirable information condition (average probability was lower than participants' rating) and events with a low estimation were more likely to be assigned to the undesirable information condition (average probability was higher than participants' rating). In this paradigm, extreme estimation in the initial session might be followed by an estimation closer to the mean at follow-up, meaning that RTM may have affected the unrealistic optimism effect in these studies.

In order to test if the RTM effect can confound these commonly cited social psychological findings, we conducted two studies using paradigms similar to those used to test the social conformity effect (Klucharev et al., 2009) and the unrealistic optimism effect (Sharot et al., 2011) in earlier research. The main difference was that participants were aware that group average rating or average probability existed, but they were given no information about these averages. Here, we used the “proof by contradiction” method to examine the effect of RTM, similar to the demonstration that statistics that were uncorrected for multiple comparisons showed active voxel clusters in the salmon's brain when the dead salmon was scanned using functional magnetic resonance imaging (fMRI) during a social perspective taking task (Bennett et al., 2009). Proof by contradiction is often used when researchers wish to prove the impossibility of something. Researchers assume it is possible, and then reach a contradiction. In the “dead salmon” example, the contradiction researchers arrive at is that “dead salmon”s brain was activated during a social task', which is obviously untrue. It proves that the statistical analysis methods used in that fMRI study is invalid. In our study, we deliberately withheld social feedback information so that no psychological effects should be found. This design is important for the present studies, because the absence of challenging information means that participants' ratings or estimations would not be influenced by experimental conditions and any change in ratings or estimations could be attributable to RTM. We first analyzed the data according to the methods used in recent studies and then analyzed it again after controlling for the RTM effect. We assumed that the RTM effect would be evident and confound the social conformity and unrealistic optimism effects even without the impact of challenging information, and we hypothesized that the social conformity and unrealistic optimism effects would not be found after RTM was controlled.

## Study 1: social conformity

### Participants

We recruited 13 healthy participants from South China Normal University (*n* = 13, four males, mean age ± SD, 21.15 ± 2.64 years). All participants were right-handed and had normal or corrected-to-normal vision and no neurological or psychiatric disorders. Written, informed consent was given by all participants and they were entitled to discontinue participation at any time. All participants were paid 30 yuan (about $5 U.S.) for their participation. The study was approved by the Ethics Committee of the School of Psychology at South China Normal University.

### Experimental paradigm

**Figure 2:**
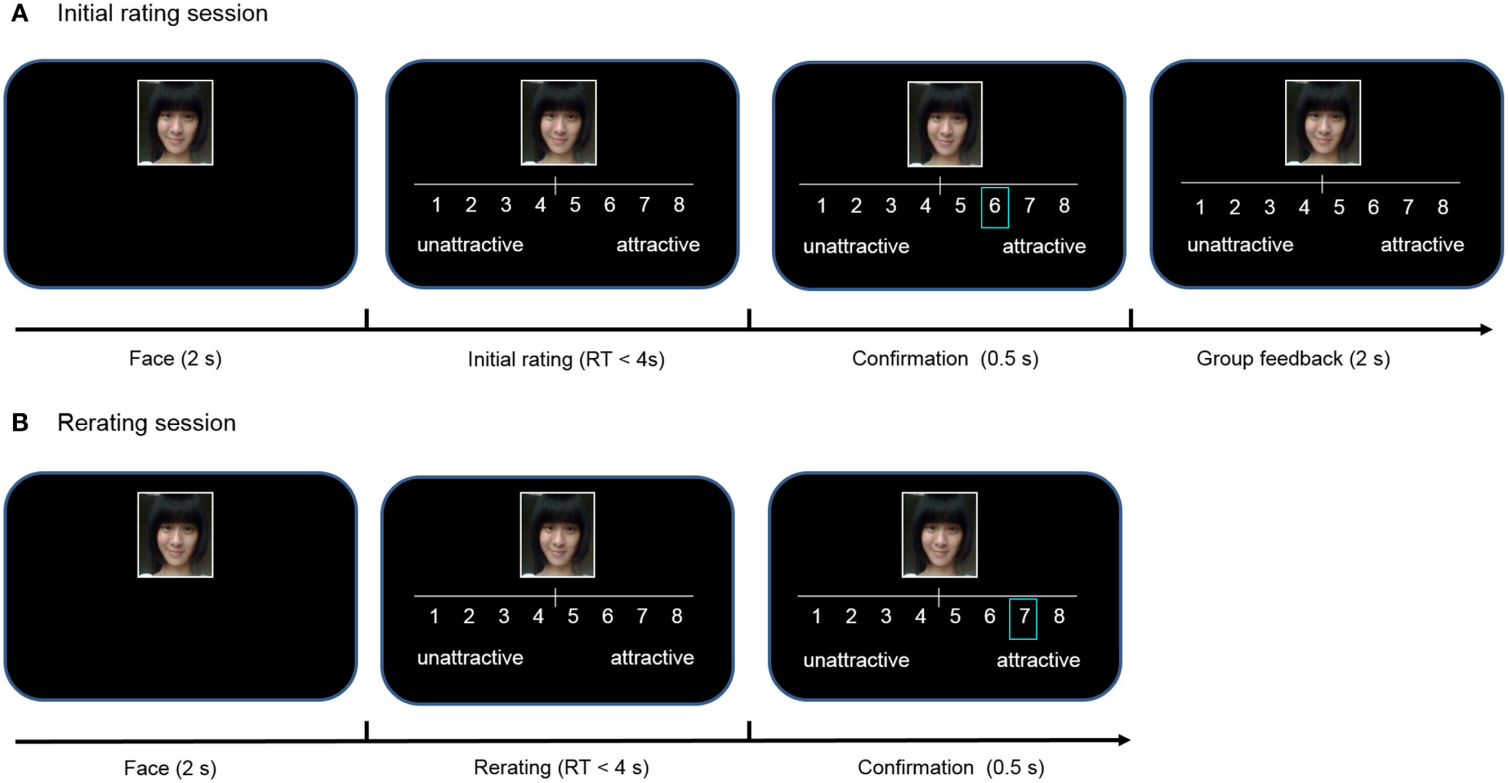
**Experimental task design in study 1. (A)** In initial rating session, a photograph of a female face was presented and participants rated face attractiveness on an eight-point Likert scale. Then, a blue box confirmed the initial rating. Finally, participants were told that group rating (calculated based on 200 other students of the same gender) was displayed within a black box. **(B)** Thirty minutes after the initial session, participants rated the same faces again in the re-rating session.

We used 280 digital photos of young adult Chinese females (Huang et al., 2014) as stimuli. Participants were informed that they were participating in a research project studying human perception of facial attractiveness. First, one of the 280 photographs of female faces was presented for 2 s. Then we instructed participants to use the mouse to rate the face on a 1 (very unattractive) to 8 (very attractive) scale in 4 s, and the number they had chosen was highlighted by a blue box for 0.5 s. Then, in order to create an experimental context similar to the Klucharev et al. (2009) study and to make participants believe that they were informed of the group rating even though they were not, participants were told that the group rating (calculated based on 200 other students of the same gender) would be displayed within a black box for 2 s but they would not be able to see it (Figure 2A). Although this phase is labeled as “group feedback” in Figure 2A, only the same initial rating was shown and no group feedback was actually provided. The absence of feedback information ensures that participants' ratings or estimations would not be influenced by experimental conditions and any change in ratings or estimations could be attributable to RTM. This manipulation is critical for our “proof by contradiction” approach as explained above.

There were 280 trials in total. The group rating programmed in the computer varied across trials. In 70 trials, the group rating matched the participant's rating (peers-agree condition). In 105 trials, the group rating was higher than the participant's rating by 1, 2, or 3 points (peer-higher condition). In the other 105 trials, the group rating was lower by 1, 2, or 3 points when compared with the participant's rating (peer-lower condition). Presentations of all face stimuli were randomized across participants and conditions.

Thirty minutes after the first behavior session, participants were required to conduct an unexpected subsequent session (Figure. 2B). In this session, 280 faces in a new randomized order were rated by participants again without information about the peer group rating. Thus, participants rated twice in the experiment: the first time before the “group rating” phase and the second time 30 min after the first session. After the entire experiment, participants indicated that they believed there had been an average group rating shown to them but it was not visible.

### Data analysis and results

At the beginning of the analysis phase, the ratings of faces in each session were mean-corrected (Sharot et al., 2012a) so as to control overall changes in ratings across sessions (Mean-Corrected Rating = Rating for a Face - Average Rating for all 280 Faces). We also computed a rating change score for each face (i.e., Mean-Corrected 30-min Re-rating - Mean-Corrected Initial Rating).

**Figure 3:**
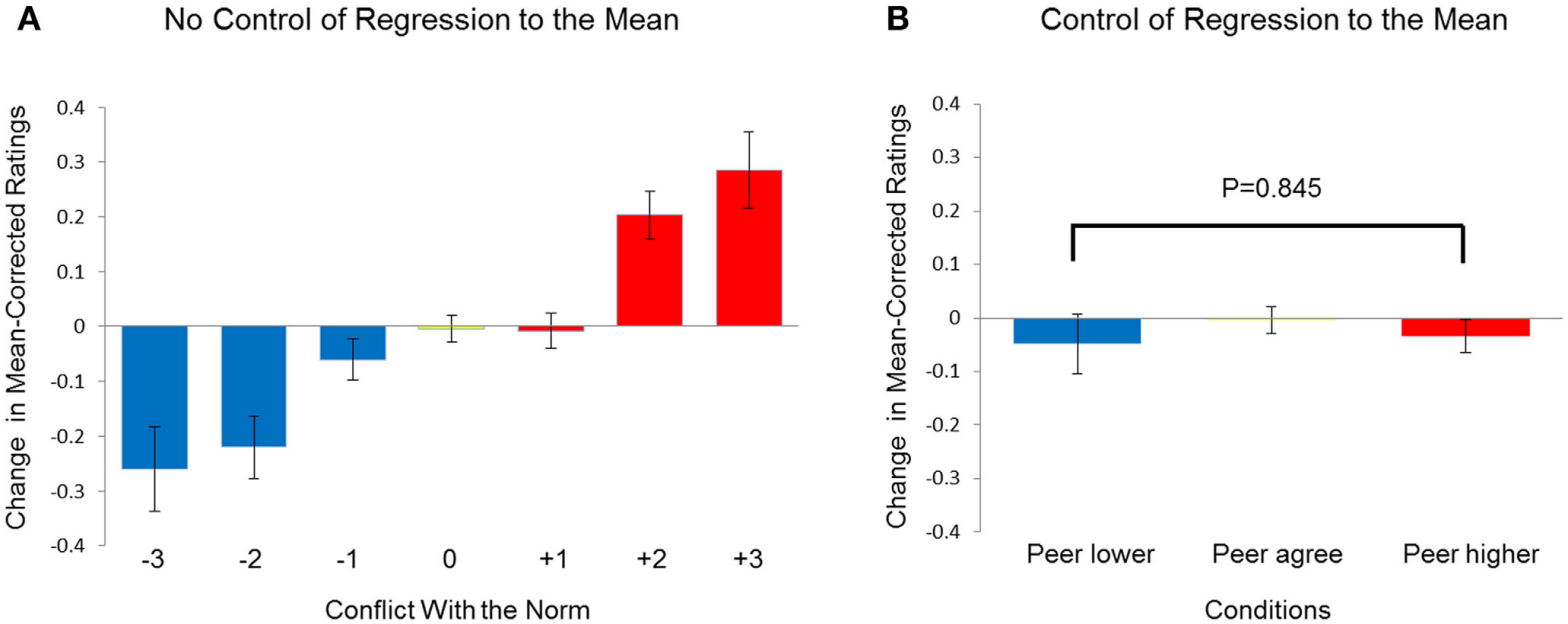
**Social conformity effect in study 1. (A)** Change in mean-corrected ratings after 30 min (mean-corrected 30-min re-rating minus the mean-corrected initial rating) as a function of conflict with the norm (−1, −2, −3 = group ratings were more negative than initial ratings by 1, 2, 3, points;; 0 = no conflict; +1, +2, +3 = group ratings were more positive than initial ratings by 1, 2, 3 points). **(B)** Change in mean-corrected ratings after 30 min as a function of conditions. To control for regression to the mean, the results were from subsets of faces with initial ratings matched between the peer-higher and peer-lower conditions. Error bars indicate standard error.

We then used the method of Klucharev et al. (2009) to analyze the behavioral data. A One-Way ANOVA on rating change scores with conflict (Group Rating - Participant's Initial Rating, with seven possible scores: ±3, ±2, ±1, 0) as a within-subject variable confirmed the significant main effect of conflict [*F*_(6, 12)_ = 15.009, *p* < 0.001], consistent with a social conformity effect. This result suggests that participants tended to align themselves with the peer-group ratings presented 30 min earlier (Figure 3A), a conclusion that is obvious impossible given that no social feedback was actually presented in the first place. This “contradiction” proves that the statistical analysis methods applied above are incorrect and the RTM effect needs to be controlled.

In order to control for the RTM effect, we analyzed the behavior data again using method of Zaki et al. (2011). At first, for each participant, we selected a subset of faces for which the participant' initial ratings were matched across the peers-lower and peers-higher conditions (see Figure 1B, left panel). For each participant, we excluded faces with initial ratings smaller than 7% in the peer-higher condition and faces with initial ratings larger than 93% in the peer-lower condition. After the exclusion, there was no significant difference between participants' initial rating in peer-higher and peer-lower condition, *t*(12) = 0.743, *p* = 0.472. The average number of trials for the peers-lower and peers-higher conditions was 71.46 (SD = 19.97) and 81.62 (SD = 12.61), respectively. The subsequent analysis only used the matched subsets data. The paired-samples *t* test on rating change scores revealed no significant difference between peers-lower and peers-higher conditions for the interval of 30 min, *t*_(12)_ = −0.199, *p* = 0.845 (Figure 3B).

Finally, regression analysis was conducted with degree of conflict as the independent variable and rating change as the dependent variable. Conflict had a significant effect on rating change, beta = 0.082, *p* < 0.001. However, when we controlled for the RTM effect by adding the initial rating as another independent variable (see Figure 1B, middle panel), the new regression model showed that conflict no longer significantly predicted the rating change, beta = 0.005, *p* = 0.54. Thus, after RTM was controlled we found no evidence of the social-conformity effect.

## Study 2: unrealistic optimism

### Participants and experimental paradigm

To ascertain whether an experimental effect other than social conformity could be confounded by the RTM effect, we next examined the unrealistic optimism effect in the absence of average probability. Fifteen healthy, right-handed participants were recruited via South China Normal University (*n* = 15, 4 males, mean age ± SD, 20.93 ± 1.75 years). All participants received a uniform payment of 30 yuan (about 5 US dollars).

**Figure 4:**
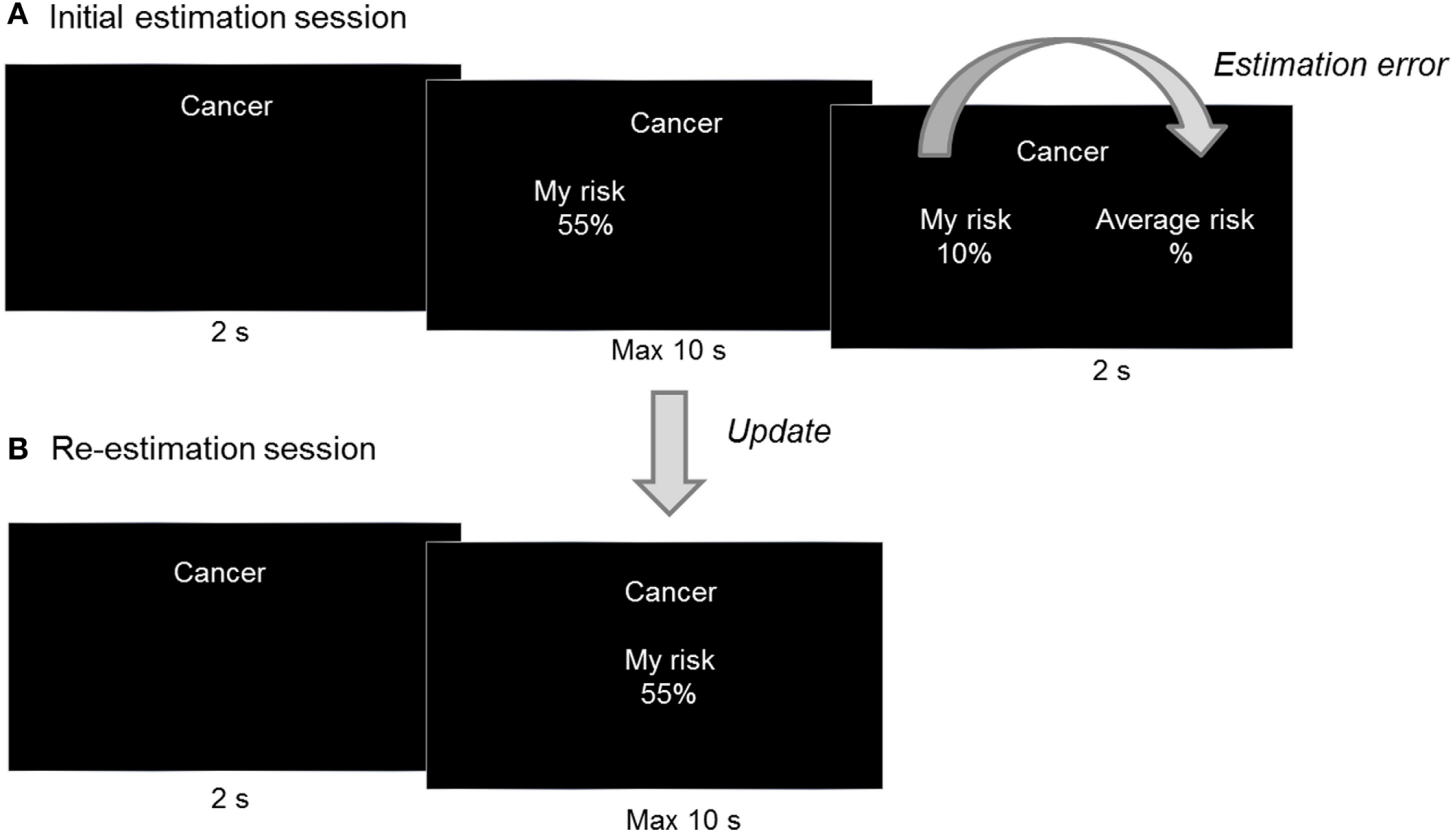
**Experimental task design in study 2. (A)** During each trial of the initial estimation session, one of 80 adverse life events was presented and participant estimated the likelihood of experiencing the event. The initial estimation was shown under “My risk.” Finally, participants were told that the average probability of that event occurring to people from background similar to them was displayed in black font under “Average risk.” For each event, the difference between “My risk” and “Average risk” was calculated as estimation error. **(B)** In the re-estimation session, participants estimated these 80 adverse life events again. For each event, the difference between the initial estimation and re-estimation was calculated as update.

Eighty short descriptions of adverse life events from the study of Sharot et al. (2011) were used as stimuli, with descriptions taking into account the Chinese context (see Supplementary List of Stimuli in Study 2). The stimuli did not include very rare or very common events, with probabilities ranging from 5% to 80%. In each trial, one of those 80 negative life events was presented at random for 2 s. Then an initial value of 55 percent was shown under “My risk” on the screen's left side and participants were asked to estimate the likelihood of experiencing the adverse event in the future by changing the initial value in 10 s. Participants were told that the range of probabilities was between 5% and 80%, which was consistent with the actual range of probabilities. This method was slightly different from that used in the study of Sharot et al. (2011) as no possible overestimation and underestimation were ensured. However, this method can help to exclude the influence of extreme estimation due to an artificially enlarged range of average probability and can facilitate an accurate test of the experimental effect using this paradigm. Specifically, we set the initial value of 55 percent so as to make it convenient for participants to adjust their estimation of experiencing adverse events upwards or downwards. Participants could increase and decrease the tens digit of the initial value by pushing F and G keys while the J and K keys could be used to control the units digit. Finally, for 2 s, participants' estimated risk of the event was shown under “My risk” while no average probability of that event occurring to people from a background similar to their own was presented under “Average risk.” But, to make the experimental context similar to the study of Sharot et al. (2011) as well as make participants believe that they were informed of the average probability, participants were told that the average probability was presented in black font under “Average risk” and they cannot see it (Figure 4A). Participants reported at the end of the experiment that they believed an average probability of each event had been presented but was invisible.

Unlike in the Sharot et al. (2011) study, in the current study no accurate average probability for each adverse event could be calculated, even with the use of online resources. Therefore, average probability in current study was programmed in the computer using the following criteria: in half the trials, the average risk was higher than the participant's estimation by 2 to 21 percentage points (undesirable condition) while in the remaining trials it was below the participant's estimation by -21 to -2 percentage points (desirable condition). After 30 min, participants took part in an unexpected subsequent estimation session identical to the first (Figure 4B).

### Data analysis and results

**Figure 5:**
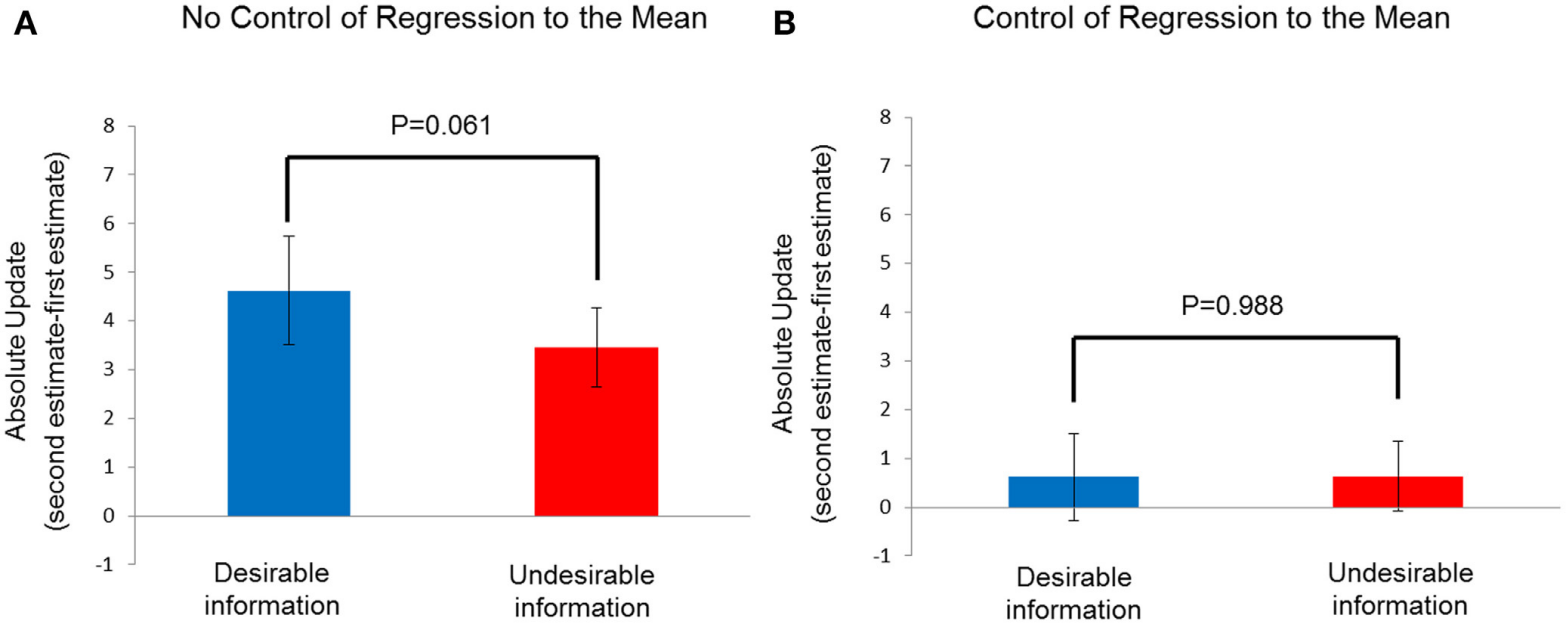
**Unrealistic optimism effect in study 2. (A)** Absolute update in mean-corrected estimations after 30 min (|mean-corrected 30-min re-estimation - the mean-corrected initial estimation|) as a function of conditions (desirable information vs. undesirable information). **(B)** Absolute update in mean-corrected estimations after 30 min as a function of conditions. The results were from subsets of events with initial estimations matched between the desirable information and undesirable information conditions, to control for regression to the mean. Error bars indicate standard error.

The mean-corrected estimation for each session was computed as (Estimation for an Event - Average Estimation for all 80 Events) and update of each event was computed as (Mean-Corrected 30-min Re-estimation - Mean-Corrected Initial Estimation). We then analyzed the behavioral data with the methods used by Sharot et al. (2011), comparing the absolute update (|Mean-Corrected 30-min Re-estimation - Mean-Corrected Initial Estimation|) in the desirable vs. undesirable condition through paired-samples *t* test. The results showed that there was a significant difference between the absolute updates seen in the desirable condition and undesirable conditions *t*_(14)_ = 2.037, *p* = 0.061. This suggests that participants are more likely to update their estimations when the information is desirable than when it is undesirable (Figure 5A), a conclusion that is obviously wrong since no feedback was actually presented.

In order to control for the RTM effect, we selected a subset of events that were matched with participants' initial estimations between desirable condition and undesirable condition. For each participant, events with initial estimations that were smaller than 13% in the undesirable condition and events with initial estimations that were larger than 77% in the desirable condition were excluded. Difference between participants' initial estimations in the desirable and undesirable conditions was not significant, *t*_(14)_ = −0.547, *p* = 0.593. The average number of trials in the desirable and undesirable conditions was 22.87 (SD = 8.03) and 29.67 (SD = 9.64), respectively. The difference in absolute update between the conditions providing desirable and undesirable information was not significant *t*_(14)_ = 0.016, *p* = 0.988, indicating that participants updated their estimation in the desirable information condition as much as in the condition of undesirable information (Figure 5B).

Using regression analysis, estimation error (Average Risk - Initial Estimation) was the independent variable while update between the two sessions was the dependent variable. The coefficient for estimation error was significant with RTM not controlled (beta = 0.318, *p* < 0.001). However, after initial estimation was used as an additional independent variable, estimation error no longer significantly predicted update scores from the 30-min retest (beta = 0.07, *p* > 0.05). Thus, there was no evidence of an unrealistic optimism effect after RTM was controlled.

## Discussion

As is common in social psychological research, earlier studies on the social conformity effect and the unrealistic optimism effect have relied on repeated measurements but have not fully controlled for the effects of RTM. In the current studies we demonstrated that the social conformity effect and unrealistic optimism effect were remarkable before controlling for RTM but were no longer apparent after controlling for RTM. Overall, our findings support the conclusions that the social conformity effect and unrealistic optimism effect can be confounded by the RTM effect if it is not controlled; to ensure valid conclusions, it is essential to control for the RTM effect in social psychology studies.

Our results are consistent with those of a recent longitudinal study on social conformity (Huang et al., 2014). In the Huang et al. study, a facial-attractiveness rating task was used and participants were asked to rate each face; they were then informed of the rating of a peer group; finally, they were called back to rate the same faces after 1, 3, or 7 days or 3 months. Their results demonstrated the importance of controlling for the RTM effect, as the social-conformity effect at the 3 months interval disappeared after RTM was controlled. Our study took this line of research one step forward. Firstly, our results showed that the RTM effect can confound the social conformity effect even when participants are not presented challenging information. Different from Huang et al.'s study, no challenging information was presented to participants and that means participants rated the same faces twice without any outside influence from experimental conditions. Therefore, any change between the two ratings should be attributed to the inside statistical problem of RTM caused by random error and extreme ratings. Secondly, the results of our study showed that an effect other than social conformity—namely, unrealistic optimism—was also confounded by RTM. This is important because no other study to date has attended to RTM in the study of unrealistic optimism. Finally, the most important point is that our findings challenge the conclusions made based on previous studies on social conformity and unrealistic optimism. In other words, it is likely that previous studies suffered from the RTM effect and those results were affected by it. Using an inert treatment (no feedback), we demonstrated a significant RTM effect which is analogous to the “placebo” effect in medicine. It is worth mentioning that our findings cannot be taken as evidence that the conformity effect and the unrealistic optimism effect examined using the test–retest paradigms are completely an artifact. Our studies differ from previous studies in many ways, including the stimuli used, the experimental parameters, culture background of participants, and so on. Moreover, there was no actual feedback provided to participants. The ambiguity induced by the lack of information may also make participants feel less confident about their ratings and thus make changes more frequently. Nevertheless, our study does suggest that the observed effects might be exaggerated if the RTM effect is not well-controlled and can be mistakenly taken as part of, if not the whole of, the psychological effect the researchers intend to examine.

Our findings also highlight the need to identify methods to control for the RTM effect in future studies of social conformity and unrealistic optimism and in the larger domain of social psychology research. As the expected change (or update) due to RTM can be mistaken for a real change (or update), it is indispensable to find ways to control for the RTM effect in order to obtain accurate results. First of all, recognizing and understanding the RTM effect is the foundation of controlling for it (Morton and Torgerson, 2005). Several methods can be applied to control for RTM. At the very least, the RTM effect should be quantified using the formula provided in previous research (Gardner and Heady, 1973; Davis, 1976; Barnett et al., 2005) that has highlighted the contribution of both within-subject variance and between-subject variance to RTM. However, this formula is not suitable for non-normally distributed data (James, 1973; McDonald et al., 1983). An even better approach to control for RTM would be to use a control group combined with random allocation of subjects at the study design stage (Whitney and Von Korff, 1992; Barnett et al., 2005). With random assignment, scores in both groups should be equally affected by RTM and the difference in mean change between the two groups should be attributable solely to the effects of the experimental manipulation (see Figure 1B, right panel).

The quantification of the RTM effect and the use of a control group can offer great protection against RTM, but RTM cannot be controlled sufficiently when extreme initial values are not excluded. As the initial value is more extreme, the expected change in the follow-up score will be greater, thus increasing the likelihood of RTM (Linden, 2013). Our present studies provide an ideal method to fully control for RTM by eliminating the impact of extreme initial values. Specifically, extreme values in the initial session were excluded so as to make sure that participants' initial ratings or estimations did not differ significantly across experimental conditions (*p* > 0.20) (Zaki et al., 2011; Huang et al., 2014). Thus, ratings or estimations in different conditions suffered from random error (and thus RTM) to the same degree. Therefore, RTM can be controlled sufficiently and the difference in change between different conditions can be attributed to the experimental effects. Here, we present evidence that experimental effects in two separate studies became statistically non-significant after eliminating the impact of extreme values in the first measurement. One disadvantage of using a subset of matched trials is that it reduces statistical power. The regression analysis which uses the complete dataset can also examine the effect of initial rating and feedback information. The results were confirmed by regression analysis conducted in both studies. In Study 1, the degree of conflict (group rating - participants' initial rating) served as the independent variable and rating change (re-rating – initial rating) was the dependent variable in the regression model. Then, to control for RTM, initial rating was included as another independent variable. The results showed that conflict could not significantly predict the change in scores after RTM was controlled. Similar results were obtained in the regression analysis conducted in Study 2. Thus, our findings demonstrate that eliminating the impact of extreme values is a successful method to fully control for RTM.

In conclusion, this is the first study in social psychology to provide an adequate test of RTM effect in absence of any feedback. Because insufficient control of RTM could lead to erroneous conclusions, social psychological researchers should pay more attention to the RTM effect in repeated measurements and adopt appropriate ways to control for it.

## Author contributions

R. Yu developed the study concept. L. Chen performed the testing and collected the data. L. Chen analyzed and interpreted the data under the supervision of R. Yu. L. Chen drafted the manuscript, and R. Yu provided critical revisions. All authors approved the final version of the manuscript for submission.

## Funding

This study was supported by the National Natural Scientific Foundation of China (Grant 31371128 to R. Yu).

### Conflict of interest statement

The authors declare that the research was conducted in the absence of any commercial or financial relationships that could be construed as a potential conflict of interest.
