# Supplementary material for: The need to control for regression to the mean in social psychology studies
Source: Front Psychol. 2015 Jan 8;5:1574. doi: 10.3389/fpsyg.2014.01574 (PMC4287101; doi:10.3389/fpsyg.2014.01574)
Supplement: Supplementary file 1 [file DataSheet1.DOCX]

The need to control for regression to the mean for repeated measurements

Rongjun Yu*, Li Chen

**Supplementary List of Stimuli in Study 2**

victim of violence by stranger

leukemia

severe anorexia

sudden flood attacks while travelling

marrying a man(woman) who is 20 years older than you

continuous blackout for more than two weeks

depression

severe hearing problems

gallbladder stones

house vandalized

being blinded

skin burn

sexual dysfunction

hepatitis A or B

epilepsy

cerebral concussion

cerebral hemorrhage

being bald because of heavy life pressure

Alzheimer’s disease

bankruptcy

joining a pyramid scheme

loss of language ability

being caught for deliberately not paying a fare

fraud when buying something on the internet

missing the train

diabetes (type 2)

alcoholism

being fired

cancer

having a stroke

victim of violence at home

asthma

tumor

laptop being stolen

pulmonary tuberculosis

remarriage

traffic accident

being extorted

heart disease

fraud from online love affair

knee osteoarthritis

domestic burglary

chronic high blood pressure

disease of spinal cord

appendicitis

rhinitis

divorce

severe insomnia

alimentary toxicosis

being cheated on by husband/wife

drunk driving

loss of ID card

privacy exposure

buying expired food

sport related accident

computer crash with loss of important data

bicycle being stolen

abnormal heart rhythm

ulcer

herpes

intention of suicide

driving through a red light

crossing the road without watching the traffic lights

getting married after age 30

in order to make a living, accepting a job that other people don’t want

flu

asking for sick leave for more than two weeks

dying before age 80

serious dental problems when old

stomachache

not being able to buy a ticket to go home during the Spring Festival

not passing the interview for a job

QQ being stolen

never going abroad during one’s whole life

being a mortgage slave

mouse in house

back pain

migraine

being harassed by phone calls from a stranger

being disappointed in a love affair
